# Supplementary material for: Stratospheric ozone loss over the Eurasian continent induced by the polar vortex shift
Source: Nat Commun. 2018 Jan 15;9:206. doi: 10.1038/s41467-017-02565-2 (PMC5768802; doi:10.1038/s41467-017-02565-2)
Supplement: Supplementary file 1 — Supplementary Information [file 41467_2017_2565_MOESM1_ESM.pdf]

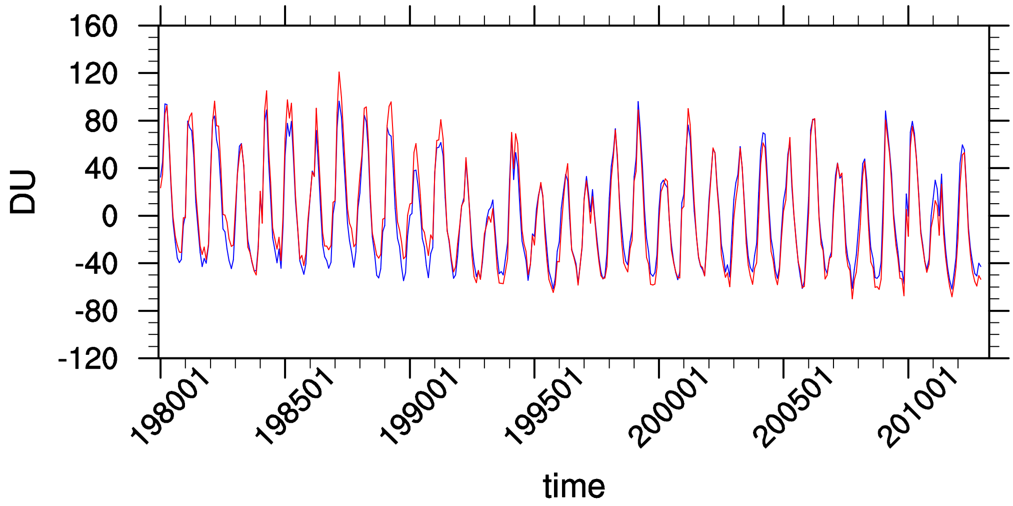

Supplementary Figure 1| Total column ozone anomaly comparison between MSR2 and SLIMCAT. Time series of monthly-mean total column ozone (TCO) anomaly (DU) with respect to climatology over the Eurasian continent for the period 1980-2012 derived from (blue) MSR2 data and (red) SLIMCAT simulation.

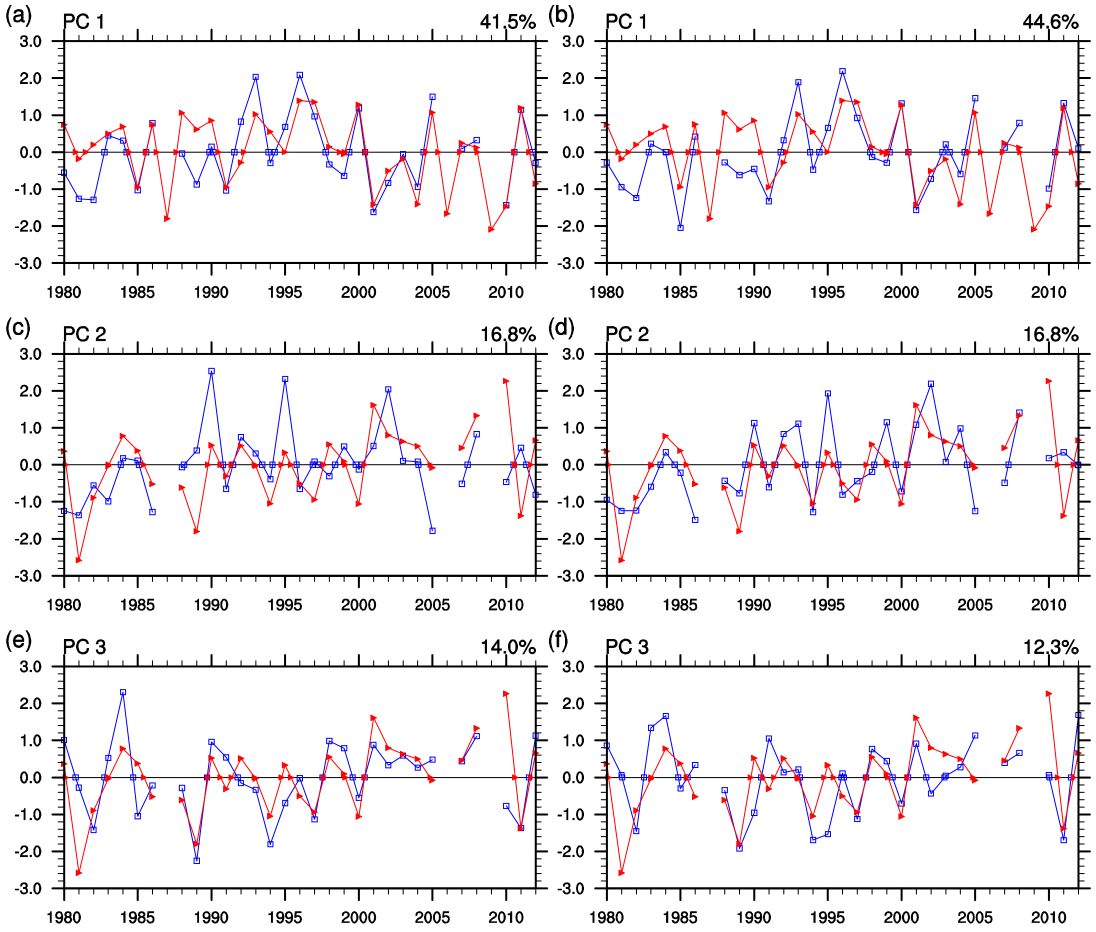

Supplementary Figure 2| Principal components of total column ozone. Time series of normalized principal component (PC) 1, PC2, and PC3 (blue lines) of February-mean total column ozone (TCO) between 45-90° N derived from **(a,c,e)** MSR2 data and **(b,d,f)** SLIMCAT simulation. The number at the top right corner of each plot represents the percentage of explained variance. Time series of polar vortex intensity (red line, 430-600 K potential vorticity averaged between 65-90° N) is overlaid in the PC1 panels. Time series of vortex shift index (see Methods section, red line) are overlaid in the PC2 and PC3 panels. The values in February 1987, 2006 and 2009 are not shown because the polar vortex broke up and its shape was distorted in these months (see Methods section).

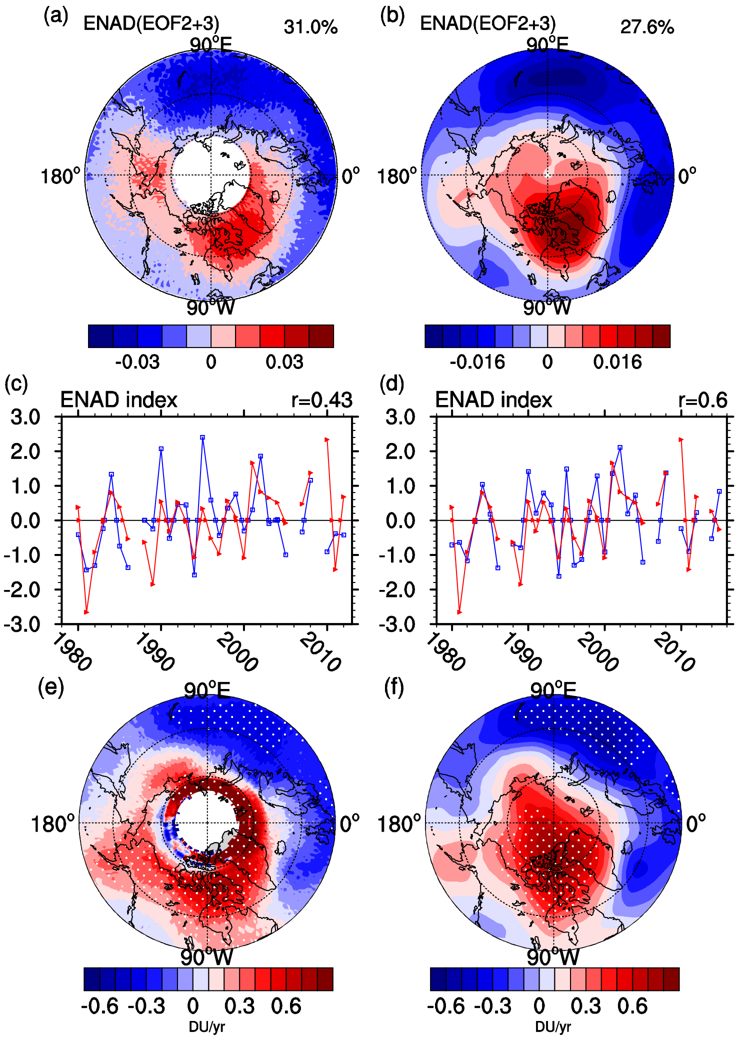

Supplementary Figure 3| Eurasia-North America dipole mode in different ozone data. (a–b) Spatial patterns of 'Eurasia-North America dipole (ENAD) mode' (EOF2+EOF3) and **(c–d)** time series of normalized ENAD index (principal component (PC)2+PC3) (blue lines) of February total column ozone (TCO) over 45–90°N derived from **(a,c,e)** NIWA data during 1980-2012 and **(b,d,f)** ERA-Interim data during 1980-2015. Time series of vortex shift index (see Methods section, red lines) are overlaid in **c–d**. The percentage of explained variance is shown in the top right of **a–b**, and the correlation coefficients between PC2+PC3 and the vortex shift index are shown in the top right of **c–d**. Linear trends of TCO regressed on the vortex shift index are shown derived from **(e)** NIWA data and **(f)** ERA-Interim data. The linear trends over the dotted regions are statistically significant at the 90% confidence level according to the Student's t-test. The minimum latitude of polar stereographic projections is 45°N. The values in February 1987, 2006, 2009 and 2013 are not

calculated because the polar vortex broke up and its shape was distorted in these months (see Methods section).

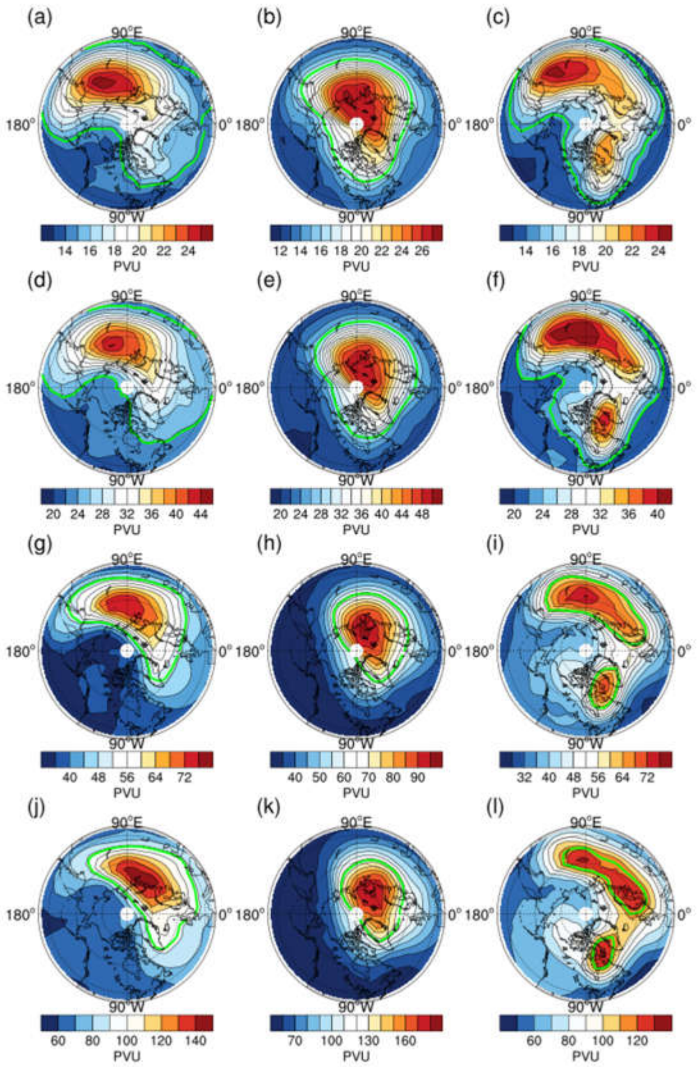

Supplementary Figure 4| Polar vortex analysis at different levels. February potential vorticity in **(a,d,g,j)** 2001, **(b,e,h,k)** 2008 and **(c,f,i,l)** 2010 on the isentropic layers **(a-c)** 430 K, **(d-f)** 475 K, **(g-i)** 530 K, and **(j-l)** 600 K. The green contour represents the edge of Arctic stratospheric polar vortex (the definition of polar vortex edge please see Methods section) at different levels.

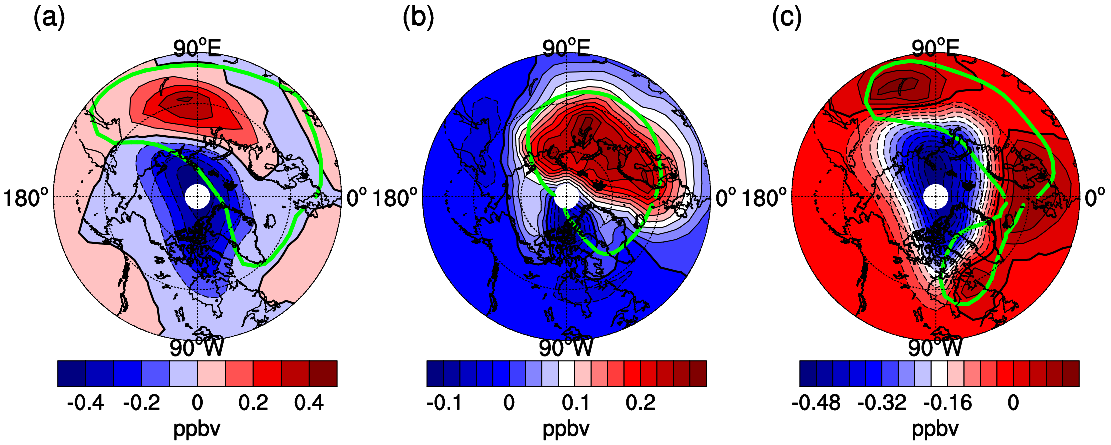

Supplementary Figure 5| ClO_x anomalies during vortex shift events. February anomalies of ClO_x in **(a)** 2001, **(b)** 2008 and **(c)** 2010 with respect to climatology averaged over the isentropic layer from 430 to 600 K. The polar vortex is shifted towards the Eurasian continent in these three years. The green contour represents the edge of the polar vortex.

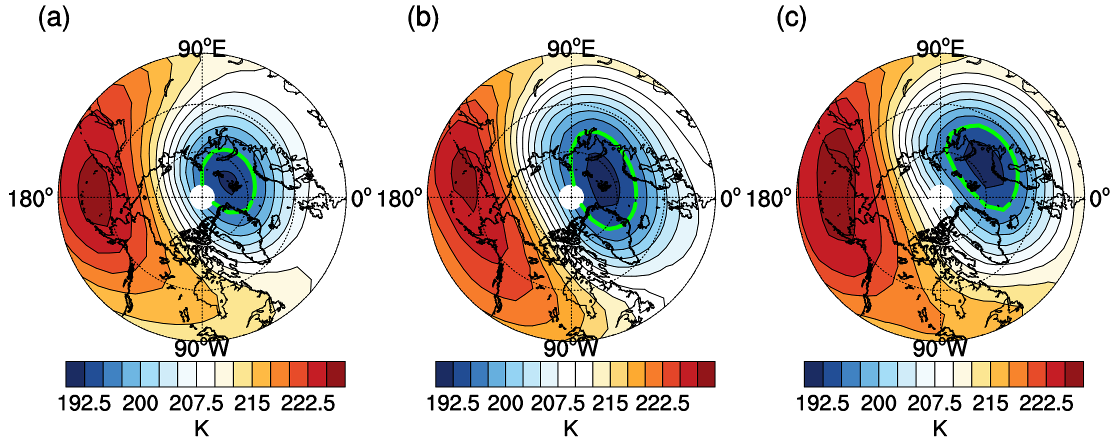

Supplementary Figure 6| Vortex temperature in January. January temperature averaged over the isentropic layer from 430 to 600 K in **(a)** 2001, **(b)** 2008 and **(c)** 2010. The green line represents the contour value of 195 K.

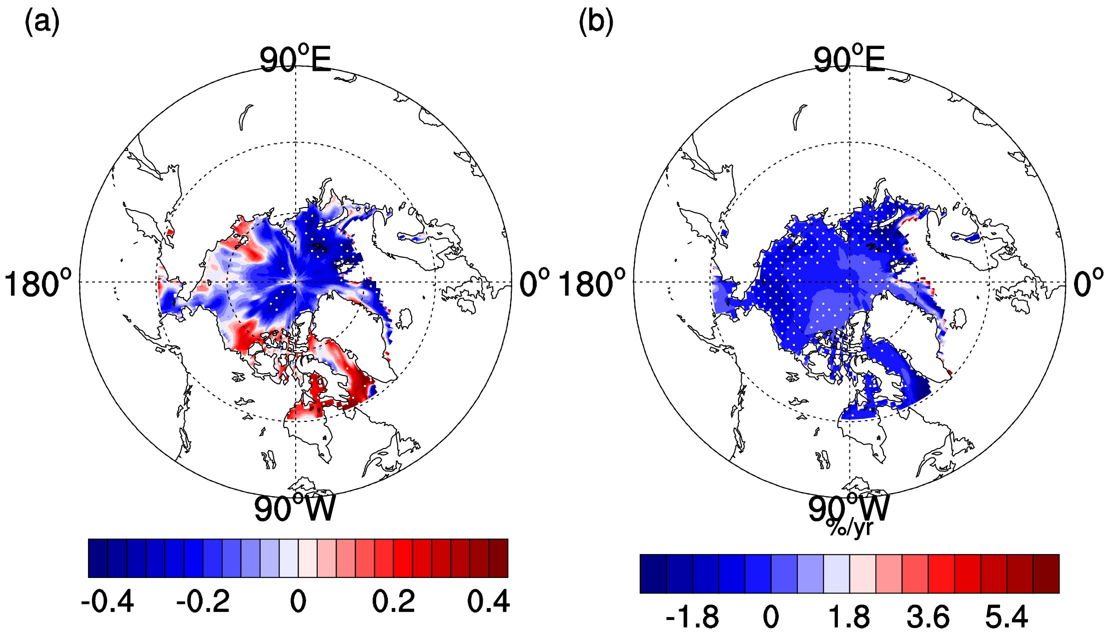

Supplementary Figure 7| Eurasia-North America dipole and sea ice. (a) Correlation coefficients between February mean Eurasia-North America dipole (ENAD) and its preceding September-October-November-December-January-February (SONDJF) mean sea ice concentration (SIC). The correlation coefficients over the dotted regions are statistically significant at the 90% confidence level according to the Student's t-test. **(b)** Linear trends of SONDJF mean percentage SIC during 1980-2012 derived from the Hadley Centre Sea Ice data set. The linear trends over the dotted regions are statistically significant at the 90% confidence level according to the Student's t-test.

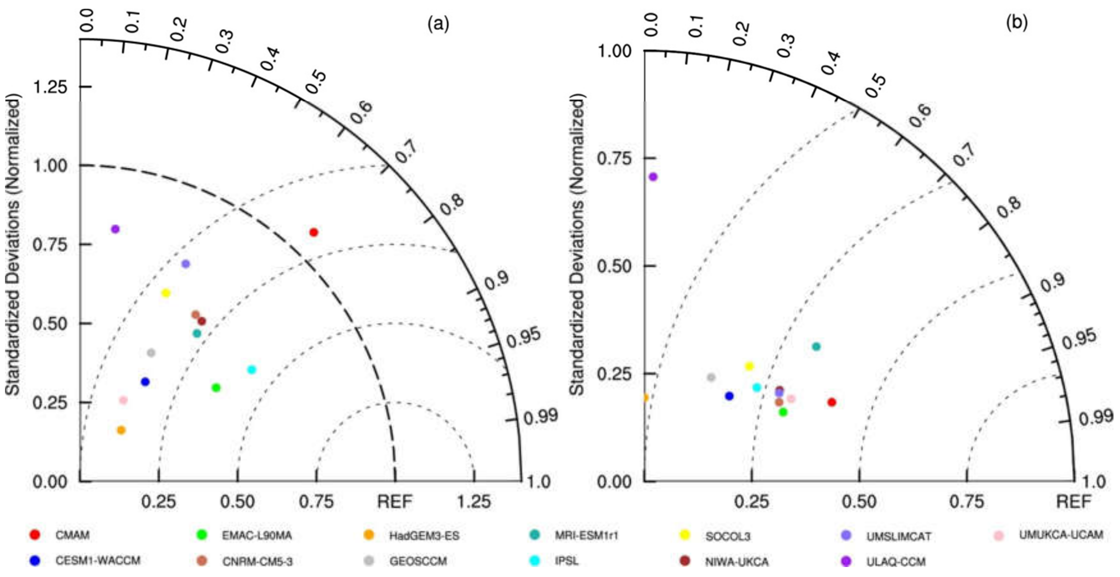

Supplementary Figure 8| EOF spatial modes of CCMI-1 REF-C1 simulation. Taylor diagram of simulated **(a)** EOF1 and **(b)** Eurasia-North America dipole **(**ENAD) derived from CCMI-1 simulations in February for period 1980-2010. Note that the ENAD in chemistry-climate model simulations is equal to EOF2 plus EOF3 when both principal component (PC) 2 and PC3 have significant correlation coefficients with the polar vortex shift index. Otherwise, ENAD only stands for one spatial pattern of either PC2 or PC3 whose correlation coefficient with vortex shift index is larger because some models fail to totally capture the similar relationships between EOF2/EOF3 and polar vortex shift as those in observation.

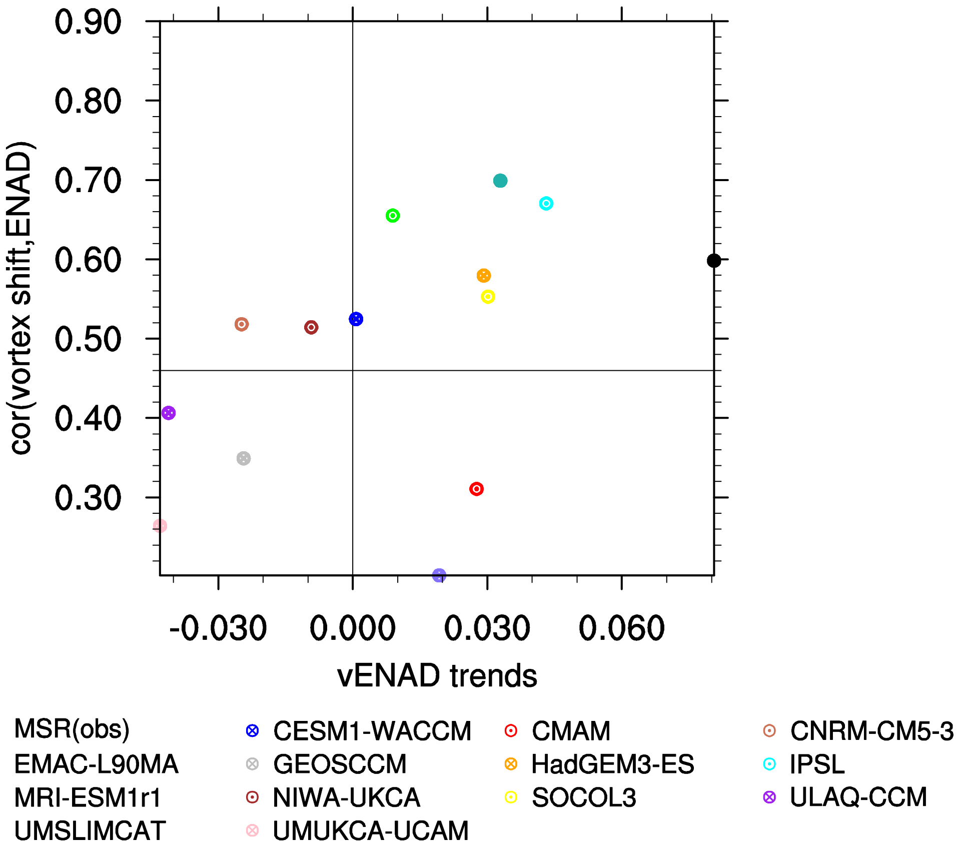

Supplementary Figure 9| Polar vortex shift and Eurasia-North America dipole of CCMI-1 REF-C1 simulation. Scatter plot of correlation coefficient between polar vortex shift and Eurasia-North America dipole (ENAD) indices, and linear trends in 'vENAD' derived from CCMI-1 REF-C1 simulations in February for period 1980-2010. The 'vENAD' is a combined measure of polar vortex shift and ENAD indices which reflects the overall performance of chemistry-climate model (CCM) in simulating the vortex shift and ENAD. It is simply equal to the sum of normalized polar vortex shift index and normalized ENAD index. The black horizontal line represents the correlation coefficient is significant at 99% confidence, while the vertical line denotes the zero value of 'vENAD' trend. Solid circle, circle with a dot and a cross inside represents the CCM of which ENAD stands for EOF2+EOF3, EOF2 and EOF3, respectively.

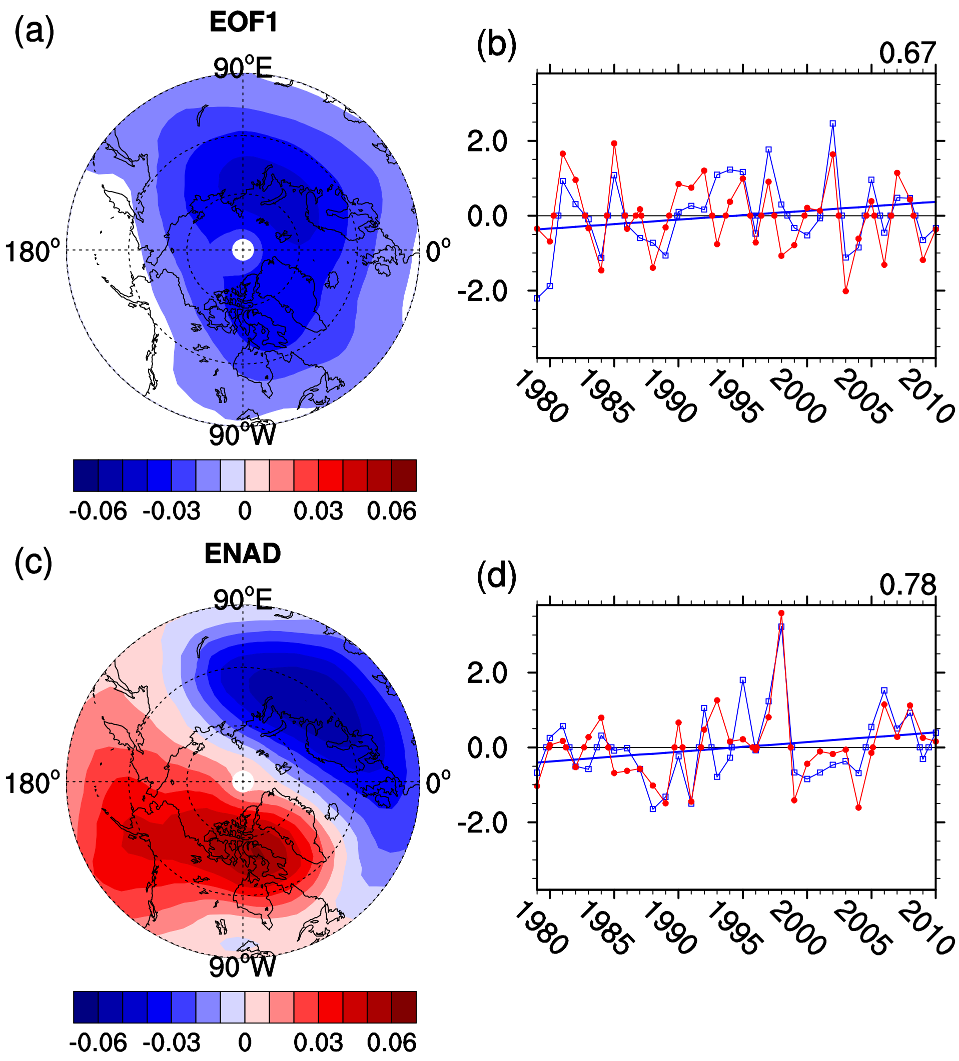

Supplementary Figure 10| Historical changes in total column ozone and polar vortex. Spatial patterns of **(a)** EOF1 and **(c)** Eurasia-North America dipole (ENAD) mode, and time series of **(b)** principal component (PC) 1 (blue curved line) and **(d)** ENAD index (blue curved line) of multi-model mean total column ozone (TCO) of EMAC-L90MA, MRI-ESM1r1 and IPSL in February. The red curved lines in **b** and **d** represent the polar vortex strength and shift index, respectively. The blue straight lines in **b** and **d** represent linear trends of PC1 and ENAD index, respectively. The correlation coefficient between PC1 and the vortex strength is shown in the top right of **b**, and correlation coefficient between ENAD index and the vortex shift index is shown in the top right of **d**.

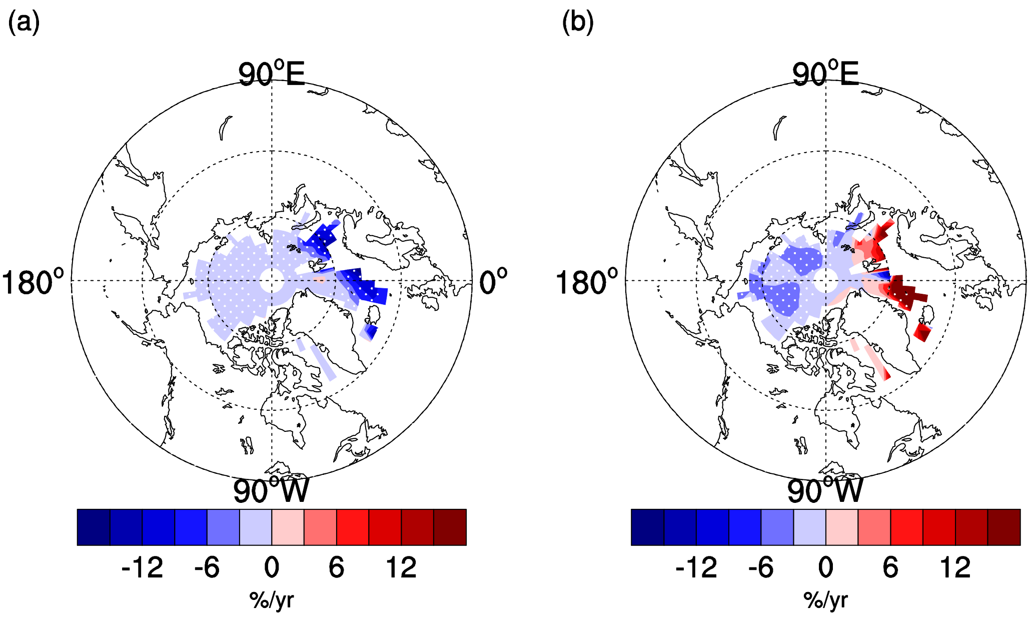

Supplementary Figure 11| Future Arctic sea-ice trends in CCMI-1 REF-C2 simulations. Linear trends by percentage in multi-model mean September-October-November-December-January-February (SONDJF) mean sea ice concentration (SIC) of EMAC-L90MA, MRI-ESM1r1 and IPSL for the period **(a)** 2010-2041 and **(b)** 2041-2050. The linear trends over the dotted regions are statistically significant at the 90% confidence level according to the Student's t-test.

Supplementary Note 1

Evaluation of CCMI-1 simulations

Taylor diagram (Supplementary Figures 8) provides a way of graphically summarizing how closely a spatial pattern from CCMI-1 REF-C1 simulations matches with observation. With regard to the EOF1 of TCO (Supplementary Fig. 8a), the spatial patterns in the simulations of 5 CCMs, i.e., IPSL, EMAC-L90MA, MRI-ESM1r1, NIWA-UKCA and CNRM-CM5-3, are most close to that in the observations (REF in Fig. Supplementary Fig. 8a). For the ENAD mode (Supplementary Fig. 8b), the spatial patterns in the simulations of the 5 CCMs still keep high correlations with the observations. Furthermore, the 5 models can also reproduce the positive correlation between vortex shift and ENAD indices. However, only IPSL, EMAC-L90MA and MRI-ESM1r1 can capture the positive trends in polar vortex shift and ENAD indices (Supplementary Figure 9) as seen in the observations. Although both HadGEM3-ES and SOCOL3 can also simulate a positive trend in the vortex shift and ENAD, they could not well reproduce the EOF1 or ENAD spatial pattern (Supplementary Fig. 8) seen in the observations. Therefore, the simulations from IPSL, EMAC-L90MA and MRI-ESM1r1 are chosen for further analysis. Supplementary Figure 10 shows the multi-model mean spatial patterns of EOF1 and ENAD mode and their time series derived from CCMI-1 REF-C1 simulations of IPSL, EMAC-L90MA and MRI-ESM1r1. The multi-model mean well captures the spatial patterns of EOF1 and ENAD modes as well as positive trends of PC1 and ENAD index in the historical period.
